# Super‐Elastic Carbonized Mushroom Aerogel for Management of Uncontrolled Hemorrhage

**DOI:** 10.1002/advs.202207347

**Published:** 2023-04-10

**Authors:** Ganghua Yang, Zhenzhen Huang, Alec McCarthy, Yueyue Huang, Jingye Pan, Shixuan Chen, Wenbing Wan

**Affiliations:** ^1^ Department of Orthopaedic Surgery The Second Affiliated Hospital of Nanchang University Nanchang Jiangxi 330006 China; ^2^ Zhejiang Engineering Research Center for Tissue Repair Materials Wenzhou Institute University of Chinese Academy of Sciences Wenzhou Zhejiang 325000 China; ^3^ Department of Surgery‐Transplant and Mary and Dick Holland Regenerative Medicine Program University of Nebraska Medical Center Omaha NE 68198 USA; ^4^ Key Laboratory of Intelligent Treatment and Life Support for Critical Diseases of Zhejiang Province The First Affiliated Hospital of Wenzhou Medical University Wenzhou Zhejiang 325000 China; ^5^ Zhejiang Engineering Research Center for Hospital Emergency and Process Digitization The First Affiliated Hospital of Wenzhou Medical University Wenzhou Zhejiang 325000 China

**Keywords:** cellulose, hemostatic materials, platelet activation, porous structure, uncontrolled hemorrhage

## Abstract

Uncontrolled hemorrhage is still the most common cause of potentially preventable death after trauma in prehospital settings. However, there rarely are hemostatic materials that can achieve safely and efficiently rapid hemostasis simultaneously. Here, new carbonized cellulose‐based aerogel hemostatic material is developed for the management of noncompressible torso hemorrhage, the most intractable issue of uncontrolled hemorrhage. The carbonized cellulose aerogel is derived from the *Agaricus bisporus* after a series of processing, including cutting, carbonization, purification, and freeze‐drying. In vitro, the carbonized cellulose aerogels with porous structure show improved hydrophilicity, good blood absorption, and coagulation ability, rapid shape recoverable ability under wet conditions. And in vivo, the carbonized aerogels show effective hemostatic ability in both small and big animal serious hemorrhage models. The amount of blood loss and the hemostatic time of carbonized aerogels are all better than the positive control group. Moreover, the mechanism studies reveal that the good hemostatic ability of the carbonized cellulose aerogel is associated with high hemoglobin binding efficiency, red blood cell absorption, and platelets absorption and activation. Together, the carbonized aerogel developed in this study could be promising for the management of uncontrolled hemorrhage.

## Graphical abstract

In this study, carbonized cellulose aerogels derived from *Agaricus bisporus* are developed for intraperitoneal hemostasis. The carbonized cellulose aerogels with porous structure show improved hydrophilicity, good blood absorption, and coagulation ability, and rapid shape recoverable ability under wet conditions. Furthermore, the carbonized aerogels show effective hemostatic ability in both in vitro and in vivo studies.

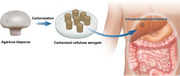

## 1 Introduction

Uncontrolled bleeding, particularly noncompressible torso hemorrhage (NCTH) and compressible junctional (limb‐torso intersection) hemorrhage (CJH), is the leading cause of trauma death in both battlefield injuries and other fortuitous accidents (e.g., road traffic crashes, occupational injuries). Given the acute nature of such injuries, timely, readily‐available, and efficacious interventions may save many soldiers and civilians who might otherwise perish.^[^ ^1^ ^]^ Death or associated complications related to uncontrolled hemorrhaging not only brings endless suffering to families, but also burdens patients’ and the government's healthcare expenditures. Compared to other acute bleeds, NCTH is generally the most difficult to address and therefore has the highest associated mortality. For example, road traffic crashes cause 1.2 million deaths globally each year, 40% of which (480 000 people) are caused by NCTH. The estimated cost is $518 billion every year. Additionally, NCTH accounts for ≈85% of preventable battlefield deaths.^[^ ^2^, ^3^ ^]^ Currently, surgery is the only way to completely treat NCTH. However, there are no point‐of‐care operational conditions at the site of the accident or on the battlefield. Therefore, many patient outcomes are determined by the duration of transit from the accident site to the hospital. While ambulances may expedite emergency transportation, rural areas and battlefield transit time can be up to 6 h.^[^ ^2^ ^]^ Therefore, highly efficacious hemostatic materials are necessary to temporarily stop bleeding and until patients are admitted to a hospital. Unfortunately, there are no satisfactory hemostatic materials for the treatment of noncompressible torso hemorrhage. Self‐expanding polyurethane foam and Xstat are some of the most promising hemostatic materials for the management of NCTH as they expand in situ, are easily deployed, and have high absorptive properties. However, polyurethane foam could theoretically create dangerously high intra‐abdominal pressure that may further tear the injured organ to cause a secondary injury during expansion.^[^ ^4^ ^]^ Xstat is approved to be used in CJH and noncompressible junctional hemorrhage.^[^ ^5^, ^6^ ^]^ However, it should not be used for the management of NCTH because of its potential safety risks on intra‐abdominal organs.^[^ ^7^ ^]^ Therefore, safer and more efficient hemostatic devices specifically for NCTH management are in urgent need.

As the Chinese and United States Department of Defense has sought new hemostatic products for NCTH, the development of novel hemostatic materials for NCTH management has been an area of renewed research interest. One of the most studied hemostatic materials are super‐elastic aerogels/sponges,^[^ ^8^ ^]^ which have high blood absorption capacity, fast blood clotting rates, easy delivery methods (injection after compression), and safe shearing force during expansion. For instance, Chen et al. developed a 3D PCL nanofiber sponge with several unique features: i) biodegradable, ii) super‐elastic and re‐expandable, iii) a high expansion ratio, iv) a nanofibrous structure, and v) ease of incorporation of hemostatic agents. The PCL sponges showed excellent hemostatic ability in a severe liver injury model and large area hepatectomy model.^[^ ^1^, ^9^ ^]^ The complex preparation processes and poor shape recovery ability after long‐term compression restricts its application. Kim et al. synthesized a catechol‐modified chitosan and utilized it to fabricate an adhesive hemostatic sponge which was inspired by wet‐resistant mussel adhesion principles. The rapid formation of an adhesive barrier at the bleed site is essential for such adhesive sponges to control hemorrhage. However, these sponges showed limited hemostatic efficiency in non‐compressible hemorrhages.^[^ ^10^ ^]^ Other reported aerogel/sponge hemostats have a variety of potential shortcomings such as containing unapproved components (e.g., graphene,^[^ ^11^ ^]^ lacking a blood coagulation factor,^[^ ^12^ ^]^ using chemically modified polymers), or were used in small mice/rats hemorrhage models or non‐critical hemorrhage models,^[^ ^13^ ^]^ and may use suboptimal delivery methods.^[^ ^14^ ^]^ The hemostatic materials for NCTH treatment should be designed with an emphasis on safety, practicality, hemostatic efficacy, and mass‐production.

**Scheme 1:**
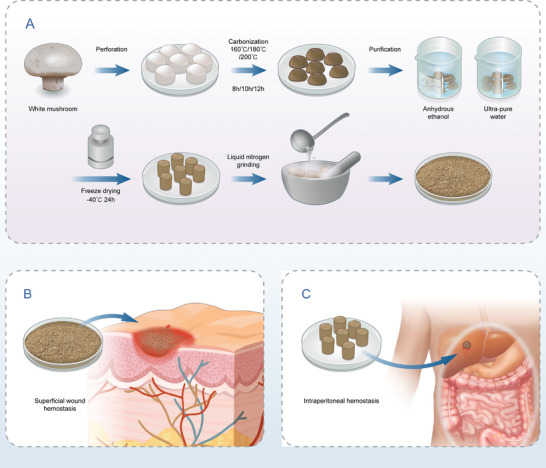
The schematic diagram is illustrating the preparation of carbonized mushroom aerogel and its application on hemostasis. A) The flow chart detailed introduces the manufacturing processes of carbonized mushroom aerogel, including cutting, carbonization, purification, freeze drying, and grinding. B) The carbonized mushroom aerogel powders are appropriate for the hemostasis of superficial wounds. C) The super‐elastic carbonized mushroom aerogel is suitable for the hemostasis of noncompressible torso hemorrhage.

Cellulose is a natural biomaterials that has been widely used as both a pharmaceutical excipient and in wound dressings.^[^ ^15^, ^16^ ^]^ Currently, cellulose is mainly derived from cotton fibers. However, the diameter of cotton cellulose is generally thick and the processability of cotton cellulose is relatively poor. As such, it is difficult to use cotton cellulose to fabricate hemostatic devices for trauma treatment or tissue regeneration. In contrast, bacterially‐derived cellulose is a potential alternative source that can be easily processed into biomaterial scaffolds and hemostats because of its flexibility.^[^ ^17^ ^]^ Currently, however, bacterial cellulose is not easily mass produced. In this study, we use *Agaricus bisporus* as a raw material whose main component is cellulose. In addition, it only takes 30–40 days from sowing to the first harvest, and growth can occur continuously for half a year. Thus, the yield of utilizable cellulose is high in a relatively short time. In this study, *Agaricus bisporus* underwent a series of processes: carbonization, purification, and freeze‐drying. Finally, the processed *Agaricus bisporus* was fabricated into powders and molded into aerogels (**Scheme** **1**). The entire process is carried out without the introduction of any toxic reagents or excipients and no bioactive molecules or mineral powders were added. We hypothesized that the carbonized cellulose powders could be used to create hemostats for superficial wounds and the carbonized aerogels could be used as hemostats for NCTH. Herein, the hemostatic capacity of carbonized *Agaricus bisporus* materials and their potential mechanisms of action are investigated.

## 2 Results

### 2.1 The Preparation and Physical Characterization of Carbonized Mushroom Aerogels

**Figure 1:**
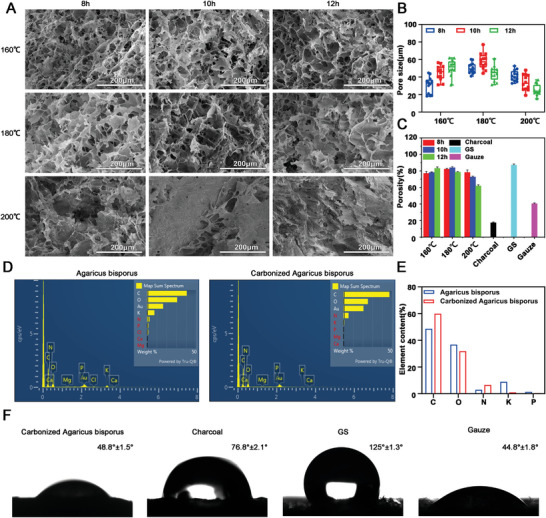
Physical characterization of carbonized mushroom aerogels. A) SEM images of the internal structure of carbonized mushroom aerogels that generated under 160 °C, 180 °C, 200 °C for 8, 10, and 12 h respectively. B) The average pore size of different carbonized mushroom aerogels. C) The porosity of the different carbonized mushroom aerogels and positive controls, including gelatin, Gauze, and charcoal. D) The EDX elemental analysis of the *Agaricus bisporus* before and after carbonization. E) The quantification of major elemental content (%) of *Agaricus bisporus* before and after carbonization. F) The contact angle of *Agaricus bisporus*, carbonized *Agaricus bisporus*, charcoal, gelatin sponge, and gauze.

The internal structure of natural *Agaricus bisporus* is fibrous (Figure S1, Supporting Information) and was turned into a porous structure after the spatial reorganization of cellulose fibers during freeze‐drying and carbonization (**Figure** **1**A). The average pore size of final carbonized aerogels carbonized under 160 °C for 8, 10, and 12 h gradually increased. The corresponding average pore size was 30.64 ± 9.49 µm, 44.81 ± 8.56 µm, and 50.99 ± 8.85 µm respectively (Figure 1B). The internal structure of the final aerogels carbonized under 180 °C with varying carbonization times still showed a porous structure (Figure 1A). The average pore size of the carbonized aerogels that generated under 180 °C for 8, 10, and 12 h were (50.24 ± 6.25) µm, (58.06 ± 9.23) µm, (44.33 ± 8.47) µm respectively (Figure 1B). However, the porous structure completely disappeared when the carbonization temperature increased to 200 °C (Figure 1A). An obvious collapsed sheet structure was shown in the carbonized aerogels generated under 200 °C for 10 and 12 h, which was similar to the charcoal structure (Figure S2, Supporting Information). The EDX elemental analysis results revealed that the content (%) of C and N elements in the carbonized aerogels increased while the content (%) of elemental O was reduced after carbonization (Figure 1D,E). The chemical functional groups of *Agaricus bisporus* before and after carbonization were identified with FTIR (Figure S3, Supporting Information) and XPS (Figure S4, Supporting Information). The hydrophilicity of the carbonized aerogels was enhanced by the presence of N‐contained chemical groups when compared with charcoal. The contact angle of the carbonized aerogel (48.8° ± 1.5°) was significantly lower than the charcoal (76.8° ± 2.1°) and gelatin sponge (125° ± 1.3°), but similar to gauze (44.8° ± 1.8°) (Figure 1F).

### 2.2 Swelling Behaviors and Shape Recoverable Ability of the Carbonized Aerogels

**Figure 2:**
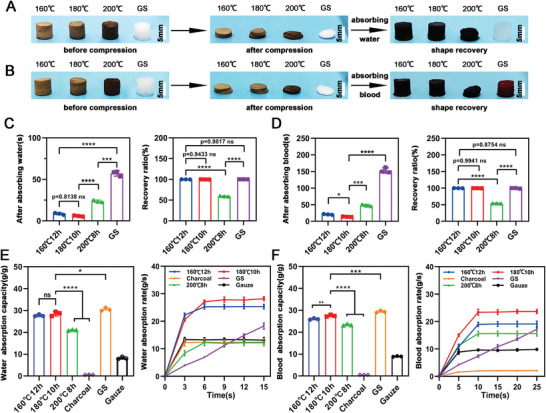
The swelling behaviors of different carbonized mushroom aerogels under water and blood. A,B) Photographs of different carbonized mushroom aerogels and gelatin sponges before and after compression, and its shape recovery behaviors after absorbing water or blood. C) The shape recovery time and shape recovery ratio of different carbonized mushroom aerogels and gelatin sponges after absorbing water (*n* = 3). D) The shape recovery time and shape recovery ratio of different carbonized mushroom aerogels and gelatin sponges after absorbing blood (*n* = 3). E) The water swelling capacity and swelling speed of different mushroom aerogels and positive controls, including charcoal, gelatin sponge, and Gauze. F) The blood swelling capacity and swelling speed of different mushroom aerogels and positive controls, including charcoal, gelatin sponge, and Gauze. *p < 0.05, ***p < 0.001, ****p < 0.0001.

We chose (160 °C, 12 h), (180 °C, 10 h), and (200 °C, 8 h) carbonized aerogels to explore shape recoverable ability under wet conditions. After absorbing water, the shape recovery time of the (160 °C, 12 h), (180 °C, 10 h), and (200 °C, 8 h) carbonized aerogels was (8.41 ± 1.20) s, (5.78 ± 0.63) s, and (23.61 ± 1.42) s respectively, which is significantly faster than gelatin sponges ((57.1 ± 3.22)s, positive control) (**Figure** **2**A,C). Further, the (160 °C, 12 h), (180 °C, 10 h) carbonized aerogels and gelatin sponges could completely recover their original shapes (Figure 2A), while the (200 °C, 8 h) carbonized aerogels only recovered (59.07 ± 0.83)% (Figure 2C). After absorbing blood, the shape recovery time of the (160 °C, 12 h) and (180 °C, 10 h) carbonized aerogels was (20.55 ± 1.35) s and (13.52 ± 0.70) s respectively, which was faster than gelatin sponges (153.28 ± 9.16) s (Figure 2B,D). The (160 °C, 12 h), (180 °C, 10 h) carbonized aerogels achieved 100% shape recovery while the (200 °C, 8 h) carbonized aerogels only recovered about 48%, and the gelatin sponge recovered about 91% (Figure 2D). Swelling capacity and swelling rate are critical parameters for hemostatic materials. As shown in Figure 2E, the (160 °C, 12 h), (180 °C, 10 h), and (200 °C, 8 h) carbonized aerogels were capable of absorbing (27.73 ± 0.49), (28.54 ± 1.04), (21.00 ± 0.22) times water, which was higher than gauze, but lower than gelatin sponges. Moreover, the carbonized aerogels showed a faster water absorption rate (less than 6 s) when compared with gelatin sponges. Finally, the (160 °C, 12 h), (180 °C, 10 h), and (200 °C, 8 h) carbonized aerogels could absorb (26.02 ± 0.33), (27.51 ± 0.43), (23.30 ± 0.39) times blood, and the (180 °C, 10 h) carbonized aerogel exhibited the fastest blood absorption (Figure 2F).

### 2.3 Super‐Elastic Mechanical Property of the Carbonized Aerogels

**Figure 3:**
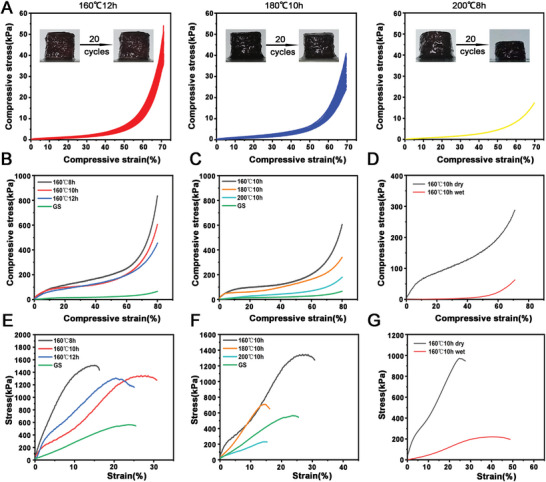
Mechanical performances of the different carbonized mushroom aerogels. A) Cyclic compression test of the carbonized mushroom aerogels that generated under 160 °C for 12 h, 180 °C for 10 h, and 200 °C for 8 h in 70% compressive strain (20 cycles) respectively. B) The compressive strain–stress curves of the gelatin sponge, and aerogels carbonized under 160 °C for 8, 10, and 12 h respectively. C) The compressive strain–stress curves of the gelatin sponge, and aerogels carbonized for 10 h under 160 °C, 180 °C, and 200 °C respectively. D) The compressive strain–stress curves of the dry and wet aerogels that carbonized under 160 °C for 10 h in 70% compressive strain. E) The tensile strain–stress curves of the gelatin sponge, and aerogels carbonized under 160 °C for 8, 10, and 12 h respectively. F) The tensile strain–stress curves of the gelatin sponge, and aerogels carbonized for 10 h under 160 °C, 180 °C, and 200 °C respectively. G) The tensile strain–stress curves of dry and wet aerogels that carbonized under 160 °C for 10 h in 30% tensile strain.

Superelasticity is one of the most important requirements for hemostatic materials in NCTH.^[^ ^1^ ^]^ As shown in **Figure** **3**A, (160 °C, 12 h) aerogel and (180 °C, 10 h) aerogel were cyclically compressed 20 times at 70% compressive strain while retaining their original shape, though the shape of (200 °C, 8 h) aerogel could not be recovered after first compression. Young's modulus and compressive stress of the aerogels carbonized under 160 °C gradually decreased with increasing carbonization time from 8 to 12 h (Figure 3B). The Young's modulus and compressive stress of aerogels carbonized for 10 h were also gradually reduced with increasing carbonized temperature from 160 to 200 °C (Figure 3C). The (160 °C, 10 h) carbonized aerogel could not only be compressed and recovered under atmospheric condition, but could also be compressed and recovered under wet condition (Figure 3D). Similarly, the tensile modulus and tensile strength of aerogels carbonized under 160 °C gradually decreased with increasing carbonization time from 8 to 12 h (Figure 3E). The tensile modulus and tensile strength of the aerogels carbonized for 10 h were also gradually reduced with the increasing carbonized temperature from 160 to 200 °C (Figure 3F). The (160 °C, 10 h) carbonized aerogel could be extended and recovered under both atmospheric and wet conditions (Figure 3G).

### 2.4 Biocompatibility of the Carbonized Aerogels

**Figure 4:**
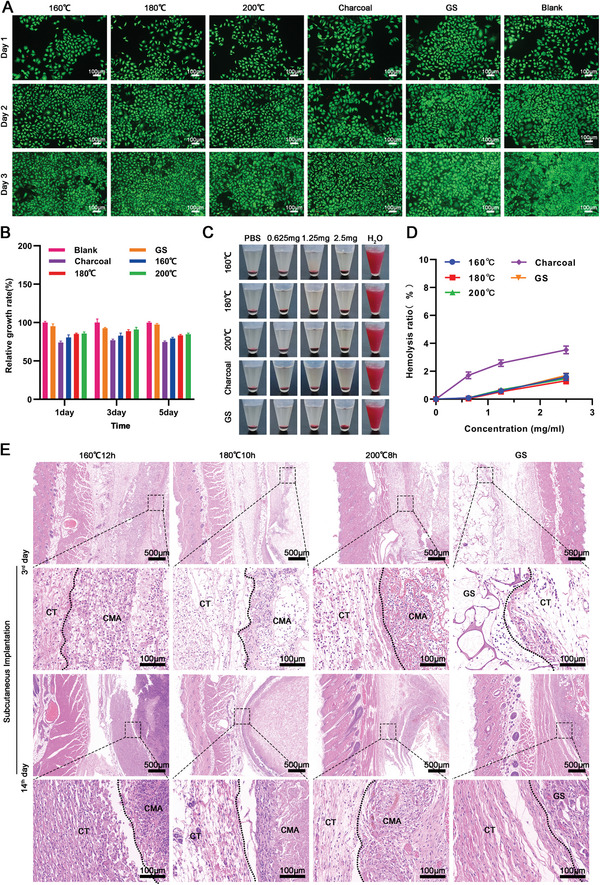
Biocompatibility evaluation of the carbonized mushroom aerogels in vitro. A,B) The proliferation and cell viability of L929 cells that co‐cultured with extract medium of different carbonized mushroom aerogels for 1, 2, and 3 days, respectively. C) The photographs of hemolysis assay of the charcoal, gelatin sponge, and carbonized mushroom aerogels with different concentrations, ranging from 2.5 to 1.25, and 0.625 mg mL^−1^. D) The quantification of the hemolysis assay. CT: connective tissues. CMA: carbonized mushroom aerogels.

The extracted liquid from the carbonized aerogels did not alter the proliferation of L929 cells (**Figure** **4**A). The relative growth rate (RGR) (%) of the (160 °C, 12 h) aerogel, (180 °C, 10 h) aerogel, and (200 °C, 8 h) aerogel were (80.58 ± 3.28)%, (85.18 ± 1.06)%, and (85.72 ± 1.99)% on day 1 (Figure 4B), which was similar with RGR at days 3 and 5. The RGR of all carbonized aerogels was higher than 75% at each time point, indicating no significant in vitro cytotoxicity according to the cytotoxicity grading criteria (ISO 10993.5‐2009). We also explored the hemocompatibility of the carbonized aerogels. As shown in Figure 4C, the charcoal group showed an obvious hemolysis phenomenon and further quantification of the hemolysis ratio (%) revealed that the hemolysis ratio (%) of (160 °C, 12 h) aerogel, (180 °C, 10 h) aerogel, (200 °C, 8 h) aerogel, and gelatin sponges were lower than the charcoal group at each indicated material's concentration (Figure 4D). Next, the in vivo biocompatibility of these carbonized aerogels was explored by subcutaneous implantation. As shown in Figure 4E, an obvious cell infiltration at the edge of the (160 °C, 12 h) aerogel, (180 °C, 10 h) aerogel, and (200 °C, 8 h) aerogel and gelatin sponges after 3 days post‐implantation were observed. By day 14 post‐implantation, more penetrated cells, new blood vessels, and collagen deposition were found within carbonized aerogels, which was similar to the positive control group (gelatin sponge).

### 2.5 In Vitro Hemostatic Efficacy of the Carbonized Aerogels

**Figure 5:**
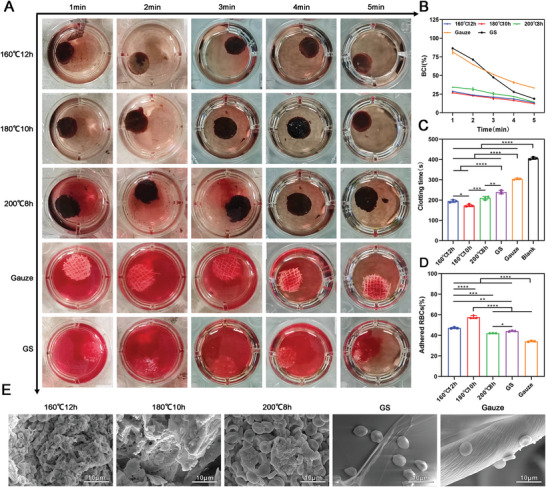
Hemostatic performances of the different carbonized mushroom aerogels in vitro. A) Photographs of hemoglobin binding capacity of the carbonized mushroom aerogels, gelatin sponge, and Gauze at different detecting times. B) The blood clotting index (BCI (%)) of the carbonized mushroom aerogels, gelatin, and Gauze at different detecting times. C) The whole‐blood clotting time of the carbonized mushroom aerogels, gelatin sponge, and Gauze. The blank group was not exposed to hemostatic materials (*n* = 3). D) The percentage of adhered RBCs on the surface of carbonized mushroom aerogels, gelatin sponge, and Gauze (*n* = 3). E) SEM images showing the interfacial reaction on the surface of carbonized mushroom aerogels, gelatin sponge, and Gauze after immersing into whole blood for 1 min. *p < 0.05, **p < 0.01, ****p < 0.0001.

First, we examined the hemoglobin binding capacity of the carbonized mushroom aerogels. As shown in **Figure** **5**A, the (160 °C, 12 h) aerogels, (180 °C, 10 h) aerogels, and (200 °C, 8 h) aerogels showed excellent hemoglobin binding efficiency when compared to the gelatin sponges, gauze. The quantified blood clotting index (BCI) (%) of three carbonized aerogels was lower than the gelatin sponges, gauze at all time points, especially in the first three minutes (Figure 5B). Notably, the (180 °C, 10 h) aerogels exhibited the fastest blood clotting relative to the other five groups (Figure 5C). Finally, we detected the absorption of red blood cells on the surface of the carbonized aerogels and found many red blood cells attached to the surface of (160 °C, 12 h) aerogels, (180 °C, 10 h) aerogels, and (200 °C, 8 h) aerogels, while few RBCs were found on the surface of the gelatin sponges, and gauze (Figure 5D,E).

### 2.6 The Adhesion and Activation of Platelets on the Surface of Carbonized Aerogels

**Figure 6:**
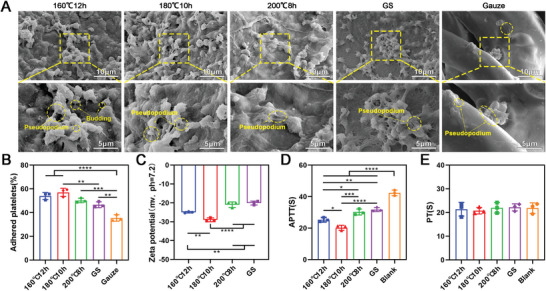
The adhesion and activation of platelets. A) SEM images showing the adhered and activated platelets (shaped changed platelets) on the surface of carbonized mushroom aerogels, gelatin sponge, and Gauze. B) Percentage of adhered platelets on the surface of carbonized mushroom aerogels, and gelatin sponge (*n* = 3). C) Zeta potential of the carbonized mushroom aerogels, and gelatin sponge (*n* = 3). D,E) Prothrombin time (PT) and activated partial thromboplastin time (APTT) analysis of the carbonized mushroom aerogels, and gelatin sponge (*n* = 3). *p < 0.05, **p < 0.01, ****p < 0.0001.

As shown in **Figure** **6**A, more platelets were found on the surface of (160 °C, 12 h) aerogels, (180 °C, 10 h) aerogels, and (200 °C, 8 h) aerogels and gelatin sponges compared to gauze. However, more platelets were attached to the surface of (160 °C, 12 h) aerogels and (180 °C, 10 h) aerogels than on the surface of gelatin sponges (Figure 6B). The shape of the activated platelets changed from round to irregular shape and contained many pseudopodiums. There was no significant difference in the numbers of attached platelets among the three carbonized aerogels groups (Figure 6B). Figure 6C revealed the zeta potential of (160 °C, 12 h) aerogels and (180 °C, 10 h) aerogels was significantly lower than the (200 °C, 8 h) aerogels and gelatin sponge. And the zeta potential of the (160 °C, 12 h) aerogels was higher than the (180 °C, 10 h) aerogels. In addition, the APTT of (160 °C, 12 h) aerogels and (180 °C, 10 h) aerogels was also lower than the (200 °C, 8 h) aerogels and gelatin sponge (Figure 6D). The blank group without any treatment (normal blood) showed the longest APTT time. There was no difference in prothrombin time (PT) among the all groups (Figure 6E).

### 2.7 Hemostatic Performance of the Carbonized Mushroom Aerogels on the Management of Rat Tail‐Amputation and Liver‐Perforation Hemorrhage Model

**Figure 7:**
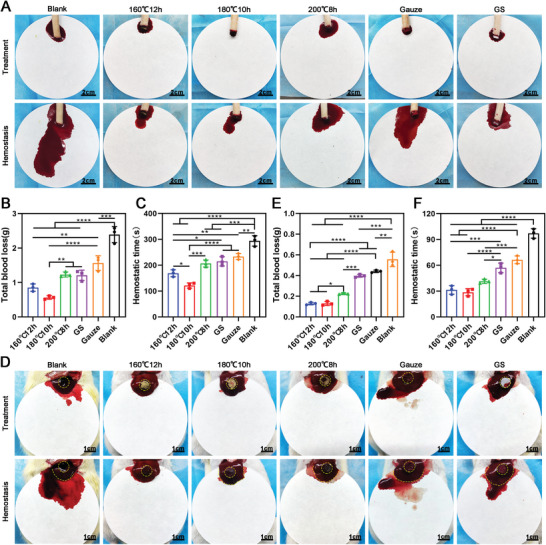
Hemostatic performance of the carbonized mushroom aerogels on the management of rat tail‐amputation and liver‐perforation hemorrhage model. A) Photographs showing the hemostatic ability of different carbonized mushroom aerogels, Gauze, and gelatin sponge in the rat tail amputation model. The tails without any treatment are set as the blank control group. B) The total blood loss of rats treated with different carbonized mushroom aerogels, Gauze, and gelatin sponge in the rat‐tail amputation model (*n* = 3). C) Hemostatic time of different carbonized mushroom aerogels, Gauze, and gelatin sponge in the rat tail‐amputation hemorrhage model (*n* = 3). D) Photographs showing the hemostatic ability of different carbonized mushroom aerogels, Gauze, and gelatin sponge in the rat liver‐perforation hemorrhage model, the perforated livers without any treatment were set as blank control group. E) The total blood loss of rats treated with different carbonized mushroom aerogels, Gauze, and gelatin sponge in the rat liver‐perforation hemorrhage model. F) Hemostatic time of different carbonized mushroom aerogels, Gauze, and gelatin sponge in the rat liver‐perforation hemorrhage model. *p < 0.05, **p < 0.01, ****p < 0.0001.

In the rat tail‐amputation hemorrhage, **Figure** **7**A,B shows the tail treated with (180 °C, 10 h) carbonized aerogels exhibited a minimum amount of blood loss (0.5643 ± 0.05) g when compared to the (160 °C, 12 h) aerogels (0.8529 ± 0.1057) g, (200 °C, 8 h) aerogels (1.232 ± 0.074) g, gelatin sponges (1.208 ± 0.1503) g, and gauze (1.563 ± 0.21) g. In addition, Figure 7C reveals the hemostatic time (121.3 ± 10.26) s of (180 °C, 10 h) carbonized aerogels was significantly faster than (160 °C, 12 h) aerogels (169 ± 13.53) s, (200 °C, 8 h) aerogels (206 ± 13.58) s, gelatin sponges (215 ± 17.69) s, and gauze (215 ± 17.69) s. Similarly, (160 °C, 12 h) aerogels and (180 °C, 10 h) aerogels exhibited the best hemostatic ability in the rat liver‐perforation hemorrhage model (Figure 7D). The blood loss in the (160 °C, 12 h) aerogel group was (0.1293 ± 0.012) g, (180 °C, 10 h) aerogel group (0.1303 ± 0.0196) g was less than the (200 °C, 8 h) aerogel group (0.2253 ± 0.0095) g, gelatin sponge (0.3980 ± 0.014) g, gauze (0.441 ± 0.012) g, and blank group (0.556 ± 0.070) g (Figure 7E). The hemostatic time of the (160 °C, 12 h) aerogels (31 ± 5.03) s and (180 °C, 10 h) aerogels (28 ± 4.16) s was less than the (200 °C, 8 h) aerogel group (41 ± 2.52) s, gelatin sponges (57 ± 5.57) s, gauze (66 ± 4.51)s, and blank group (97 ± 5.57) s (Figure 7F).

### 2.8 Hemostatic Performance of the Carbonized Mushroom Aerogels on the Management of Rabbit Cardiac Perforation and Rat Hepatectomized Hemorrhage Model

**Figure 8:**
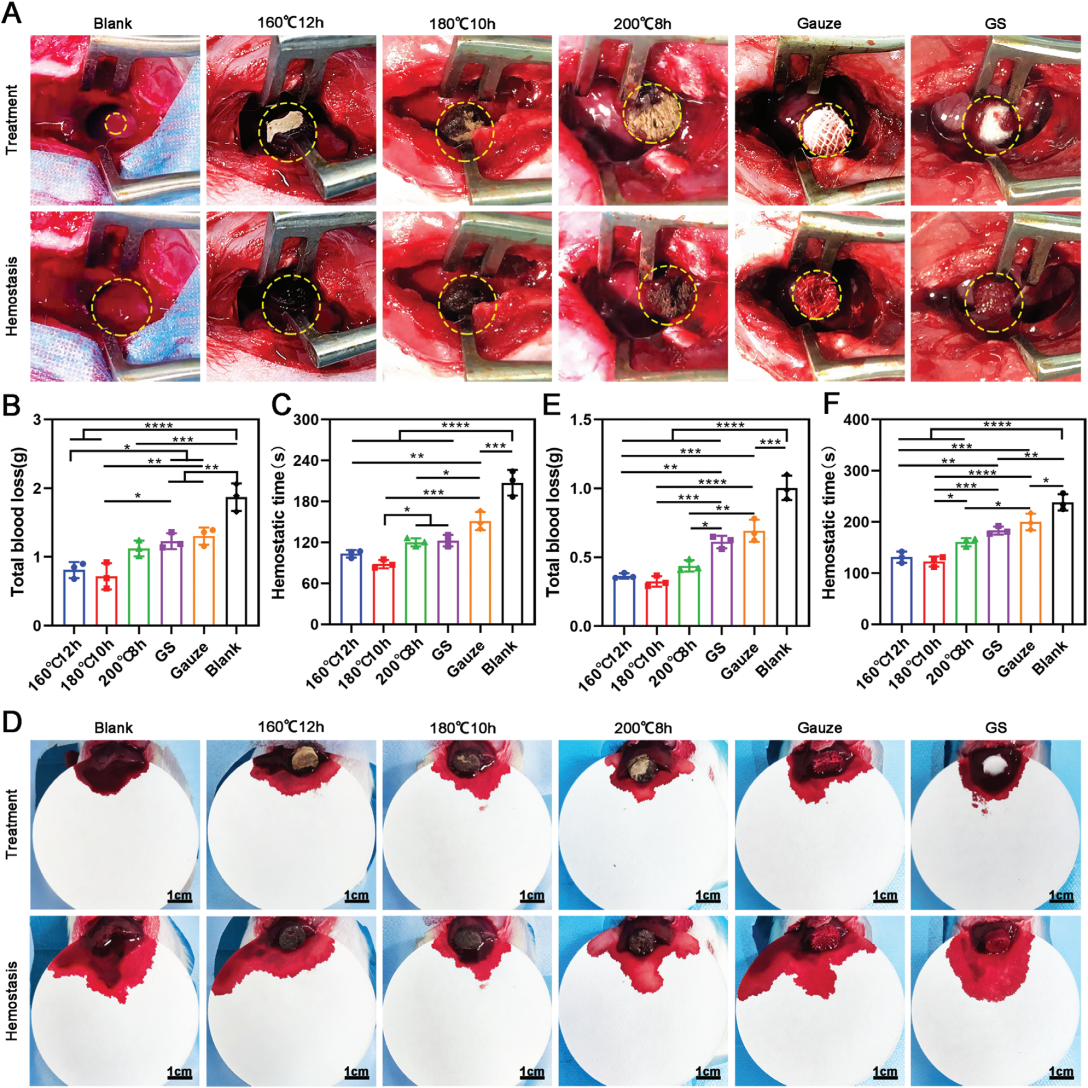
Hemostatic performance of the carbonized mushroom aerogels on the management of rabbit cardiac perforation and rat hepatectomized hemorrhage model. A) Photographs showing the hemostatic ability of different carbonized mushroom aerogels, Gauze, and gelatin sponge in the rabbit heart uncontrolled hemorrhage model. The heart without any treatment are set as the blank control group. B) The total blood loss of rabbit treated with different carbonized mushroom aerogels, Gauze, and gelatin sponge in the rabbit heart uncontrolled hemorrhage model. C) Hemostatic time of different carbonized mushroom aerogels, Gauze, and gelatin sponge in the rabbit heart uncontrolled hemorrhage model. D) Photographs showing the hemostatic ability of different carbonized mushroom aerogels, Gauze, and gelatin sponge in the rat hepatectomized hemorrhage model. The livers without any treatment were set as blank control group. E) Total blood loss of rat treated with different carbonized mushroom aerogels, Gauze, and gelatin sponge in the rat hepatectomized hemorrhage model. F) Hemostatic time of different carbonized mushroom aerogels, Gauze, and gelatin sponge in the rat hepatectomized hemorrhage model. *p < 0.05, **p < 0.01, ***p < 0.001, ****p < 0.0001.

In the rabbit cardiac perforation hemorrhage model (**Figure** **8**A), heart blood loss with the (160 °C, 12 h) aerogels and (180 °C, 10 h) aerogels was (0.81 ± 0.11) g and (0.72 ± 0.19) g, which was less than the (200 °C, 8 h) aerogel group (1.12 ± 0.12) g, gelatin sponges (1.23 ± 0.11) g, gauze (1.30 ± 0.13) g, and blank group (1.87 ± 0.2) g (Figure 8B). Further, the hemostatic time of the (180 °C, 10 h) aerogel treated group (88.33 ± 6.1) s was less than the (200 °C, 8 h) aerogel group (120 ± 6) s, gelatin sponges (122.67 ± 8.50) s, gauze (151.33 ± 13.32) s, and blank group (207.33 ± 18.77) s (Figure 8C). In the rat hepatectomized hemorrhage model (Figure 8D), liver blood loss in groups treated with (160 °C, 12 h) aerogels, (180 °C, 10 h) aerogels, and (200 °C, 8 h) aerogels was less than the gelatin sponges (0.61 ± 0.05) g, gauze (0.69 ± 0.08) g, and blank group (0.94 ± 0.16) g (Figure 8E). Finally, the hemostatic time of the (160 °C, 12 h) aerogels (133.70 ± 15.18) s and (180 °C, 10 h) aerogels (126.00 ± 14.53) s was less than the gelatin sponges (179.00 ± 12.12) s, gauze (200.00 ± 16.09) s, and blank group (238.30 ± 15.95) s (Figure 8F).

**Figure 9:**
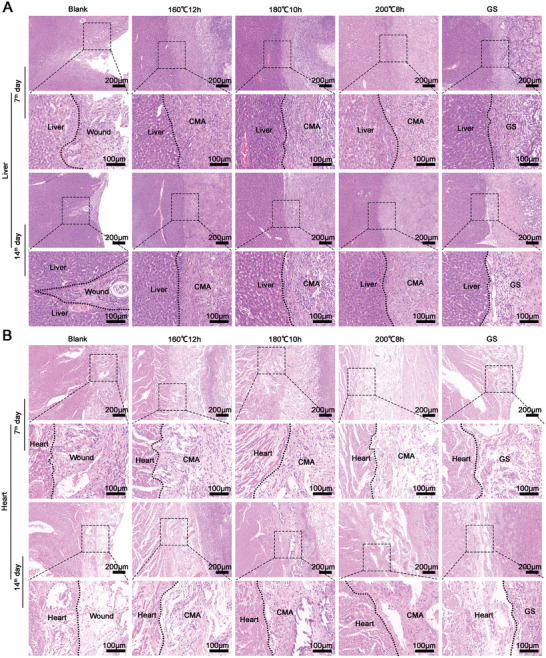
Histological observations of the injured site after achieving hemostasis. Histological observations of the interfaces between hemostatic materials and A) liver and B) heart tissues after applying them to stop bleeding for 7 days and 14 days. The black dash line refers to the boundary of the injured tissues and the hemostatic materials. CMA: carbonized mushroom aerogels.

All rats and rabbits were maintained after surgery. All rats survived in all groups after 7 days and 14 days post‐treatment. About 33% of rabbits died in the blank group, and there no dead rabbits were found in the three carbonized mushroom treated groups and gelatin foam treated group. Furthermore, histological examinations were performed after achieving hemostasis for 7 days and 14 days. As shown in **Figure** **9**, significant damaged liver and heart tissues were found in the blank group (without any treatment). Significant interfaces were found between the gelatin foam and liver tissues in the gelatin foam treated group, even after 14 days post‐treatment. While tight connections were found between the liver tissues and carbonized mushroom aerogels, especially after 14 days post‐treatment, dense fibrosis tissues were found within the carbonized mushroom aerogels (Figure 9A). In the rabbit hemorrhage model (Figure 9B), obvious loose connections between the heart tissue and (200 °C, 8 h) aerogel/gelatin sponge. However, the created holes were filled with fibrosis tissue that formed within the carbonized aerogels, and an improved connection between the heart tissues and fibrosis tissues were found in (160 °C, 12 h) aerogels, and (180 °C, 10 h) aerogels. Moreover, fibrosis tissue around carbonized aerogels and gelatin foam were found in the damaged area of liver and heart. For one thing, the human body will recognize the transplanted biomaterials as foreign bodies and produce a certain rejection reaction. A fibrous capsule will form around the transplanted biomaterials. For another, the damaged liver and heart tissue cannot be repaired immediately, and it can be temporarily replaced with fibrous tissue. Liver has strong ability to regenerate, so the damaged liver tissue can be repaired. However, cardiomyocytes are terminally differentiated cells that do not have the ability to proliferate, the damaged heart tissue can only be replaced by fibrous tissue.

## 3 Discussion

Cellulose is the most abundant organic polymers on Earth and can be widely obtained from a range of sources, including plants, algae, tunicates, and some special bacteria.^[^ ^15^, ^18^ ^]^ The main component of cellulose is polysaccharides, which consist of a linear chain of thousands of d‐glucose units. Further, the biodegradation, porosity, and interconnectivity of cellulose are adjustable.^[^ ^16^ ^]^ Therefore, cellulose can be further chemically modified by oxides, chlorides, and acids to achieve desired functions.^[^ ^19^ ^]^ Cost‐effective cellulose and its derivatives have attracted considerable attention for applications in tissue engineering,^[^ ^15^ ^]^ wounds management^[^ ^20^ ^]^ and drug delivery.^[^ ^21^ ^]^

In this study, we found that cellulose obtained from *Agaricus bisporus* has ideal processability, making it more convenient than isolates from woods and plants. The final carbonized cellulose aerogels had porous structures resulting from freeze‐drying.^[^ ^22^ ^]^ The porous structures are capable of facilitating blood absorption and forming blood clots on the interface between wounds and carbonized aerogels, leading to rapid hemostasis.^[^ ^1^, ^23^ ^]^ In addition, the hydrophilicity of the carbonized cellulose aerogels was significantly enhanced following carbonization due to introduction of N‐contained chemical groups. Improving hydrophilicity can contribute to increased blood absorption speed.^[^ ^24^ ^]^ Importantly, the carbonized aerogels exhibited excellent in vitro and in vivo biocompatibility. Pelling et al. explored the biocompatibility of plant‐derived cellulose biomaterials by subcutaneously implantation in mice. The histological observations discovered a thicker fibrous capsule developed around the cellulose after subcutaneous implantation, though cell infiltration was only found on the surface of cellulose, even after 8 weeks of implantation.^[^ ^25^ ^]^ In this study, there was no obvious fibrous capsule at the interface between carbonized aerogel and subcutaneous connective tissue. Moreover, the entire carbonized aerogels were fully infiltrated with cells by 14 days.

The delivery method of NCTH hemostatic materials differs from conventional hemostatic materials as the injection is the most accurate method to deliver hemostatic materials into the abdominal bleeding site through a small wound or incision.^[^ ^26^ ^]^ Injecting such materials requires the materials to have super‐elastic and shape‐recoverable properties.^[^ ^1^ ^]^ In other words, it is crucial to be able to compact a mass of hemostatic materials in vitro, load them into a minimally invasive device (e.g., syringe, catheter) for injection, deliver them, and have the compressed hemostatic materials immediately return to their original shape. The reported carbonized aerogels could be cyclically compressed at least 20 times and completely recover their original shape under wet conditions,^[^ ^27^ ^]^ especially in the blood.^[^ ^1^, ^28^ ^]^ The repeated compression test can fully verify the superelasticity of the carbonized aerogels, which is more reliable than one compression test. Some hemostatic materials show good shape recoverable ability in water, but recover slowly in the blood since blood has a higher viscosity than water (because it contains many blood cells, platelets, fibrinogen). Suboptimal hemostatic materials will absorb these cellular components upon contact with blood and the coagulation reaction will produce a certain resistance to shape recovery of the hemostatic material.

Biosafety is another important character of hemostatic materials for NCTH. For example, the expansile polyurethane foam can generate dangerously high intraabdominal pressures (≈100 mm Hg) during the injection.^[^ ^29^, ^30^, ^31^ ^]^ And the strong mechanical property and limited expansion space of polyurethane foam can easily cause a secondary injury to the injured organs.^[^ ^29^, ^30^, ^31^ ^]^ The Xstat is not for use in the pleural cavity, mediastinum, thorax, abdomen, retroperitoneal space, and sacral space,^[^ ^6^, ^32^ ^]^ but Tactical Combat Casualty Care (TCCC) did not specify the reason. By comparison, our carbonized cellulose aerogel does not expand unrestrictedly like polyurethane foam. The volume change is between 4 to 5 times (Figure 2B), thus it will not create dangerous intraabdominal pressures, compress other organs, or tear the original wound. In addition, we did not find side effects such as strangulation and adhesion in the in vivo studies.

The hemostatic ability is the most important performance of the presented carbonized aerogel. The carbonized aerogels are able to reduce about 79%, 76%, 67%, and 58% of blood loss in the rat tail‐amputation, liver‐perforation, and lobectomy hemorrhage models,^[^ ^9^ ^]^ rabbit cardiac perforation hemorrhage model respectively.^[^ ^33^ ^]^ Especially, the (160 °C, 12 h) aerogels and (180 °C, 10 h) aerogels exhibited the fastest coagulation rates and the least blood loss compared to the positive control group (gelatin sponges). Similarly, Wang et al. also used cellulose as a raw material and developed a porous carboxymethyl cellulose sponge, which has exhibited similar hemostatic ability in the femoral artery injury model of swine.^[^ ^34^ ^]^ The potential mechanism is related to two aspects. For one thing, the blood clotting index (BCI) of the carbonized aerogels (Figure 5B) was significant low than the gauze of gelatin foam. Suggesting that carbonized aerogels have high efficiency of hemoglobin binding and absorption ability of red blood cells.^[^ ^35^ ^]^ For another, the carbonized aerogels could significantly promote platelet absorption and activation (Figure 6A), resulting in a fast activation of the coagulation cascade in carbonized aerogel treated group.^[^ ^36^ ^]^ In this study, we speculate that the coagulation mechanism of carbonized aerogels is associated with their negative charges. Because negative charges could remarkably boost intrinsic blood coagulation by activation of coagulation factors XI and XII, along with cofactors HWK‐kininogen and prekallikrein.^[^ ^37^, ^38^ ^]^ The carbonized (160 °C, 12 h) aerogels and (180 °C, 10 h) aerogels have more negative charges, resulting in a lower APTT(s) and fast blood coagulation.

## 4 Conclusion

New carbonized cellulose‐based aerogel hemostatic material is developed for the management of noncompressible torso hemorrhage. In vitro, the carbonized cellulose aerogels with porous structure show improved hydrophilicity, good blood absorption, and coagulation ability, and rapid shape recoverable ability under wet conditions. Finally, the carbonized aerogels showed effective hemostatic ability in both in vitro and in vivo studies. Future studies may aim to enhance shape recoverability by introducing graphene oxide,^[^ ^11^, ^39^ ^]^ cryogel^[^ ^40^ ^]^ or other shape memory materials. Future studies may also aim to enhance such aerogel's hemostatic ability by making the material positively or negatively charged or b mixing with hemostatic powders (e.g., kaolin, zeolite). The cellulose and hybrid cellulose hemostatic materials are promising for the management of NCTH.

## 5 Experimental Section

### Materials

The mushroom and charcoal used in all experiments were purchased from the Yonghui Supermarket (*Agaricus bisporus*, Guangzhou, China). Gelatin sponges were purchased from Quick Health Medical Technology (catalog number: 2640494, Guangzhou, China). Dulbecco's modified eagle medium (DMEM, catalog number: 11965118), penicillin‐streptomycin (PS, catalog number: 15140122), and fetal bovine serum (FBS, catalog number: 10099–141C) were ordered from Invitrogen (CA, USA). Cell Counting Kit‐8 (CCK‐8) was purchased from Beyotime Biotechnology (catalog number: C0040, Shanghai, China), and the Acridine Orange/Ethidium Bromide Staining Kit was ordered from Mairuida Technology (catalog number: M247364, Shenzhen, China). Sodium citrate was ordered from Aladdin (catalog number: C38585, Shanghai, China). Liquid nitrogen was purchased from Ruixin Liquefied Gas Company (catalog number: XT‐175001, Tangshan, China). Calcium chloride was purchased from Alfa Aesar Chemical (catalog number: 012316, Shanghai, China). Lactate Dehydrogenase Assay Kit (LDH) was purchased from Nanjing Built (catalog number: A020‐2‐2, Nanjing, China). Glutaraldehyde was purchased from Alfa Aesar Chemical (catalog number: A17876, Shanghai, China). Ethanol was ordered from Sinopharm Chemical (catalog number: 10009228, Beijing, China). Activated Partial Thromboplastin Time kit (APTT) was purchased from Zeye Biotechnology (catalog number: ZY602874B, Shanghai, China). The Prothrombin Time kit (PT) was purchased from Sun Biotechnology (catalog number: H301, Shanghai, China). Paraformaldehyde was ordered from Aladdin (catalog number: 30525‐89‐4, Shanghai, China). Paraffin was purchased from Sigma‐Aldrich (catalog number: 8002‐74‐2, MO, USA). The Hematoxylin & Eosin staining kit (H&E) was purchased from Zeye Biotechnology (catalog number: ZY61872FA, Shanghai, China).

### Synthesis of the Carbonized Mushroom Aerogels

A one‐pot hydrothermal reaction was used to prepare the carbonized mushroom aerogels. The *Agaricus bisporus* was cut into an appropriate size and placed into a stainless steel autoclave with a tetrafluoroethylene liner (LC‐KH‐5, Lichen). The autoclave was put into an oven (P70D20TL‐D4, Galanz) and carbonized under 160 °C, 180 °C, and 200 °C for 8, 10, and 12 h respectively. The carbonized aerogel‐like products were then immersed in DI water and ethanol for two days, respectively. The super‐elastic carbonized mushroom aerogel was finally acquired after freeze‐drying at −20 °C for 24 h. The freeze dryer was purchased from Labconco company (#710201000).

### Physical Characterization of the *Agaricus bisporus* and Carbonized Mushroom Aerogels

The internal structure of the carbonized mushroom aerogels that were generated under 160 °C, 180 °C, 200 °C for 8, 10, and 12 h respectively, were observed by a field emission scanning electron microscope (FEI Nova NanoSEM 450). The zeta potential of carbonized mushroom aerogel was determined by a Nano particle size and zeta potential analyzer (DLS, Malvern Zetasizer Nano ZS90). The elemental analysis spectrum of the *Agaricus bisporus* before and after carbonization was obtained by an energy dispersive spectroscopy (Oxford Inca Energy X‐Max20). The functional groups of the A*garicus bisporus* before and after carbonization were determined in the range of 4000–500 cm^−1^ by using a Fourier transform infrared (FTIR, Thermo Scientific Nicolet 6700). The element content of the *Agaricus bisporus* before and after carbonization was detected by a X‐ray photoelectron spectroscopy (XPS, Thermo Scientific K‐Alpha, USA). The water contact angle of the aerogel surface was obtained using a surface tension/contact angle measurement instrument (Chengde Dingsheng Testing Machine Testing Equipment Co., Ltd. JY‐82 Co.).

### The Porosity and the Average Pore Size of Carbonized Mushroom Aerogels

The microstructure of the different carbonized mushroom aerogels were characterized by a field emission scanning electron microscopy (FEI Nova NanoSEM 450). The average pore size of different carbonized mushroom aerogels was measured using ImageJ. The porosity of the different carbonized mushroom aerogel was obtained using the ethanol substitution method. The initial mass of the dry aerogel was weighed as *W*_1_ (g) and the volume was measured as *V* (cm^3^). The dry aerogel was completely immersed in ethanol and removed at saturation. The excess ethanol residue on the aerogel was wiped away using filter paper. The wet aerogel was reweighed as *W*_2_ (g). The porosity of carbonized mushroom aerogels was calculated using the following equation:

(1) $$\mathrm{Porosity}\;\left(\%\right)=\left(W_{2}-W_{1}\right)/\rho V\times 100\%$$

where *W*_1_ is the weight of the dry aerogel and *W*_2_ is the weight of the wet aerogel after immersion in ethanol. *V* is the volume of the dry aerogel, and *ρ* is the density of ethanol (0.785 g cm^−3^).

### Cyclic Compression and Shape Recovery Performance

The different carbonized mushroom aerogels were prepared into cylindrical shapes with a height of 5 mm and a diameter of 5 mm. The original height of the aerogel was recorded as *H*_1_. The aerogel was compressed and squeezed out of the water to achieve shape fixation, and the height was set as *H*_2_. Next, the shape‐fixed aerogel was immersed in water or fresh whole blood (obtained from rats) to recover its shape, and the recovery height was measured as *H*_3_. The shape recovery process was recorded by a digital camera. The shape recovery time was recorded by a timer. The shape recovery ratio was calculated using the following equation:

(2) $$\mathrm{Shape}\;\mathrm{recovery}\;\mathrm{ratio}=\left(H_{3}-H_{2}\right)/\left(H_{1}-H_{2}\right)\times 100\%$$

where H_1_ is the original height of carbonized mushroom aerogel, *H*_2_ is the height of the aerogel after compression, and *H*_3_ is the height of the aerogel after absorption.

The mechanical property of carbonized mushroom aerogel was evaluated by compression testing and the cyclic compression test using an electromechanical universal testing machine (CMT6104, Mester Industrial). For the cyclic compression test, a drop of water was added around the carbonized mushroom aerogel on the platform and the wet aerogel was compressed to 70% strain, then released to 0% strain at a constant compression and release strain rate of 2 mm min^−1^. This cycle was repeated 20 times. For the compression test, the compressive strain and speed were fixed at 70% and 2 mm min^−1^, respectively. For the tensile test, the carbonized mushroom aerogel was placed on the testing machine after absorbing water and stretched at a constant rate of 2 mm min^−1^ until the scaffold was broken.

### Absorption Behaviors

To quantitatively assess the swelling behaviors of carbonized mushroom aerogels, dry aerogels with a height of 5 mm and diameter of 5 mm were first weighed as *M*_1_ (g). Afterward, the dry aerogels were immersed in water or blood and were taken out from the liquid at different detecting times and weighed as *M*_2_ (g). The swelling capacity and absorption rate were calculated according to the following equation:

(3) $$\mathrm{Water}/\mathrm{blood}\;\mathrm{absorption}\;\mathrm{capacity}\left(g\;g^{-1}\right)=\left(M_{2}-M_{1}\right)/M_{1}$$

(4) $$\mathrm{Water}/\mathrm{blood}\;\mathrm{absorption}\;\mathrm{rate}\;\left(g\;s^{-1}\right)=\left(M_{2}-M_{1}\right)/\mathrm{time}$$

where *M*_1_ is the weight of the dry aerogel, and *M*_2_ is the weight of the wet aerogel after immersion in water or blood.

### Cytotoxicity Assay

L929 (mouse connective tissue) cells were cultured in complete growth media (DMEM containing 10% FBS and 1% PS) at 37 °C with 5% CO_2_ and the influence of the carbonized mushroom aerogels on cell viability and proliferation were evaluated by CCK‐8 and AO/EB staining assays. The extracts were obtained by immersing carbonized mushroom aerogels in the growth medium overnight. L929 were seeded into a 96‐well plate at a density of 2000 cells per well and cultured at 37 °C with 5% CO_2_ for 12 h. Then the culture medium was replaced by the extract medium. After co‐culturing for 1, 3, and 5 days, CCK‐8 reagent was added into each well and further cultured at 37 °C for 2 h. The absorbance value was read at 450 nm by a microplate reader (*n* = 4). The relative growth rate were calculated according to the following equation:

(5) $$\begin{array}{ccc} \mathrm{The}\;\mathrm{relative}\;\mathrm{growth}\;\mathrm{rate}\;\left(\%\right) & = & {\mathrm{OD}}_{\mathrm{sample}}/\mathrm{Average}({\mathrm{OD}}_{\mathrm{blank}\;1}+{\mathrm{OD}}_{\mathrm{blank}\;2}\\ & & +\;{\mathrm{OD}}_{\mathrm{blank}\;3}+{\mathrm{OD}}_{\mathrm{blank}\;4}) \end{array}$$

where OD_sample_ is the absorbance value of the L929 treated with the extract medium from the carbonized mushroom aerogels, and OD_blank_ is the absorbance value of the L929 without any treatment. Additionally, L929 cells were seeded into a 24‐well plate with a cell density of 5000 cells per well and cultured for 12 h. Then the culture medium was replaced by the extract medium and co‐cultured for 72 h. The living/dead L929 cells were observed by AO/EB staining using a fluorescent microscopy (Leica DMi8).

### Hemolysis Assay

Whole rat blood was mixed with 109 mm sodium citrate at 9:1 and centrifuged at 3000 rpm for 5 min to obtain erythrocytes. The erythrocytes were washed with PBS and diluted to a concentration of 5% v/v suspension. Dry aerogels were ground into powder using liquid nitrogen to obtain the aerogel dispersion liquids with different concentrations, ranging from 2.5 to 1.25 and 0.625 mg mL^−1^. Next, 0.5 mL of aerogel dispersion with varying concentrations and 0.5 mL of erythrocytes were added into a 1.5 mL tube and mixed thoroughly. All tubes were incubated at 37 °C for 1 h and centrifuged at 10 000 rpm for 10 min. A volume of 200 µL of supernatant was carefully transferred into a 96‐well plate. The absorbance of the supernatant at 562 nm was read using a microplate reader (S/N 3052431277, Molecular Devices). The samples treated with DI water and PBS were set as positive control and negative control, respectively. The hemolysis ratio of carbonized mushroom aerogel was calculated using the following equation:

(6) $$\mathrm{Hemolysis}\;\mathrm{ratio}\left(\%\right)=\left(A_{m}-A_{p}\right)/\left(A_{h}-A_{p}\right)\times 100\%$$

where *A*_m_ represented the absorbance value of the samples, and *A*_h_ and *A*_p_ represented the absorbance value for DI water and PBS, respectively. Each group was repeated three times.

### Blood Clotting Test

The different carbonized mushroom aerogels were prepared into cylindrical shapes with a height of 5 mm and a diameter of 5 mm. Gauze, gelatin sponges, and carbonized mushroom aerogels with fixed shapes were placed into a 24‐well plate, respectively. 10 m of citrated blood was mixed with 1 m of 0.1% calcium chloride to obtain recalcified blood. A volume of 50 µL of recalcified blood was rapidly added onto the surface of gauze, gelatin sponges, and carbonized mushroom aerogels. After incubation at 37 °C for 1, 2, 3, 4, and 5 min respectively, 2 mL of DI water was added into each well to release and lyse the unbound red blood cells. The supernatant of the hemoglobin solution was transferred into a 96‐well plate, and the absorbance value was read at 562 nm. The blood‐clotting index (BCI) was calculated according to the following equation:

(7) $$\mathrm{BCI}=\left({\mathrm{OD}}_{1}-{\mathrm{OD}}_{3}\right)/\left({\mathrm{OD}}_{2}-{\mathrm{OD}}_{3}\right)\times 100\%$$

where OD_1_ represented the absorbance of the samples, OD_2_ represented the absorbance of DI water, and OD_3_ represented the absorbance of PBS.

### Hemostatic Performance In Vitro

The different carbonized mushroom aerogels were prepared into cylindrical shapes with a height of 5 mm and a diameter of 5 mm. Gauze, gelatin sponges, and carbonized mushroom aerogels with fixed shapes were placed into a 5 mL tube. 250 µL of citrate whole blood and 25 µL of 0.1% m CaCl_2_ solution were prewarmed at 37 °C for 30 min and mixed gently. The recalcified blood was added onto the surface of gauze, gelatin sponges, and carbonized mushroom aerogels. The tubes were continually turned upside down every 3 s to see if the clot formed. The hemostatic time was recorded immediately when the blood clot formed.

### Blood Cell and Platelet Adhesion

Red blood cell (RBC) suspension was obtained by centrifuging whole citrate blood at 3000 rpm for 10 min. The different carbonized mushroom aerogels were prepared into cylindrical shapes with a height of 5 mm and a diameter of 5 mm. Gauze, gelatin sponges, and carbonized mushroom aerogels with fixed shapes were placed into a 24‐well plate and a volume of 50 µL of RBC suspension was dropped on the surface of each sample. After incubation at 37 °C for 1 h, each sample was washed with PBS three times to remove non‐adherent RBCs and attached RBCs were lysed with 2 mL of DI water to release hemoglobin. A volume of 100 µL of the supernatant was introduced into a 96‐well plate and the absorbance value was measured at 562 nm using a microplate reader. The absorbance value of 50 µL of RBC suspension mixed with 2 mL of DI water was used as a reference value. The percentage of adherent red blood cells was calculated by the following equation:

(8) $$\mathrm{Percentage}\;\mathrm{of}\;\mathrm{red}\;\mathrm{blood}\;\mathrm{cell}=\left({\mathrm{OD}}_{\mathrm{sample}}/{\mathrm{OD}}_{\mathrm{reference}}\right)\times 100\%$$

For platelet adhesion, platelet‐rich plasma (PRP) was separated from citrate whole blood via centrifugation at 3000 rpm for 10 min. 50 µL of PRP was dropped on the surface of gauze, gelatin sponges, and carbonized mushroom aerogels and incubated at 37 °C for 30 min. All samples were rinsed three times with PBS to remove physically adherent platelets and then immersed in 1% Triton X‐100 to lyse platelets and release lactate dehydrogenase (LDH). The concentration of LDH was assessed using a Lactate Dehydrogenase Assay Kit. Finally, the absorbance value of the supernatant from each sample was measured at 490 nm and referred to as OD_sample_. The absorbance value of PRP not exposed to hemostatic materials was measured at 490 nm for a reference value. The percentage of adherent platelets was calculated by the following equation:

(9) $$\mathrm{Percentage}\;\mathrm{of}\;\mathrm{adherent}\;\mathrm{platelets}=\left({\mathrm{OD}}_{\mathrm{sample}}/{\mathrm{OD}}_{\mathrm{reference}}\right)\times 100\%$$

To observe the interfacial reaction on the surface of hemostatic materials, PRP was introduced onto the surface of gauze, gelatin sponges, and carbonized mushroom aerogels and incubated at 37 °C for 1 h. All samples were washed three times with PBS, fixed in 2.5% glutaraldehyde for 2.5 h, and gradually dehydrated with ethanol. The dried samples were characterized by a scanning electron microscope. For whole blood experiments, 50 µL of whole blood was introduced onto the surface of Gauze, gelatin sponges, and carbonized mushroom aerogels and incubated at 37 °C for 1, 2, and 3 min, respectively. All samples were washed three times with PBS, fixed in 2.5% glutaraldehyde for 2.5 h, and gradually dehydrated in ethanol. The dried samples were observed using a scanning electron microscope.

### APTT Assay

Activated partial thromboplastin time (APTT) assay was performed to evaluate the intrinsic coagulation system and several modifications were made to investigate the activation of the coagulation cascade response induced by the hemostatic materials. Platelet‐poor plasma (PPP) suspension was obtained by centrifuging whole citrate blood at 3000 rpm for 10 min. The contact activator for FXII in the commercial APTT kit was replaced by powders from gelatin, and carbonized mushroom aerogels ground under liquid nitrogen. PPP and all reagents were warmed up to 37° C. The phospholipids were mixed with powders from Gauze, gelatin, and carbonized mushroom aerogels gently and incubated with 1 mL of PPP at 37 °C. The final concentration of the powder from the hemostatic materials was 1 mg mL^−1^. 0.025 m CaCl_2_ was added to the mixture with an equal volume to initiate coagulation and the time was recorded.

### PT Assay

Prothrombin time (PT) analysis was conducted to investigate the role of the hemostatic materials in exogenous coagulation pathways. Powders from gelatin, and carbonized mushroom aerogels were homogeneously dispersed into PPP to form the powder suspensions with concentrations of 1 mg mL^−1^. The powder suspensions were incubated at 37 °C for 3 min. PT reagent was also pre‐warmed at 37 °C and 0.2 mL of the PT reagent was added into 0.1 mL of the powder suspensions. The clotting time was recorded.

### Hemostatic Performance In Vivo

All animal experiments were approved by the ethical committee of the Second Affiliated Hospital of Nanchang University. All animals used in the experiments were purchased from Tianqin Biotechnology Company. The hemostatic ability of different carbonized mushroom aerogels, gauze, and gelatin sponges were assessed using the rat tail‐amputation hemorrhage model, rat liver‐perforation hemorrhage model, rabbit heart uncontrolled hemorrhage model, and rat liver uncontrolled hemorrhage model. The carbonized mushroom aerogels were prepared into cylindrical shapes with a height of 8 mm or 10 mm and a diameter of 5 mm.

For the rat tail‐amputation hemorrhage model, rats were randomly divided into six groups. The animals were anesthetized by injecting 3% pentobarbital sodium and fixed on an operating table (2022042004A, Zhongke Huida Technology). A pre‐weighed filter paper was placed underneath the tail which was cut off 4 cm from the root using surgical scissors (SSJ‐16, Vogel Oriental Technology). The preweighed Gauze, gelatin, and carbonized mushroom aerogels with fixed shapes were applied to the wound immediately and the total blood loss of rats treated with different carbonized mushroom aerogels, Gauze, and gelatin sponges in the rat‐tail amputation model was recorded. The hemostatic time of the materials in the rat tail‐amputation hemorrhage model was also recorded. The tails without any treatment are set as the blank control group.

For the rat liver‐perforation hemorrhage model, the rats were randomly divided into six groups. All animals were anesthetized by injecting 3% pentobarbital sodium and then fixed on an operating table. The liver of rats was exposed by an abdominal incision. A pre‐weighed filter paper was placed beneath the liver. A circular perforated wound with a diameter of 6 mm was created on the liver to induce bleeding and the preweighed carbonized mushroom aerogels, Gauze, and gelatin sponges with fixed shapes were used to fill the wound cavity. The total blood loss and hemostatic time were recorded. After implantation for 7 days and 14 days, the injured liver was collected for histological analysis.

For the rat hepatectomized hemorrhage model, the rats were randomly divided into six groups. All animals were anesthetized by injecting 3% pentobarbital sodium and then fixed on an operating table. The liver of the rats was exposed by an abdominal incision and half of the right liver lobe was removed. Afterward, the preweighed carbonized mushroom aerogels, Gauze, and gelatin sponges with fixed shapes were immediately applied to the injured site to stop bleeding. The total blood loss and hemostatic time were recorded. Livers without any treatment were set as blank controls. After implantation for 7 days and 14 days, the injured liver was collected for histological analysis.

For the rabbit cardiac perforation hemorrhage model, the rabbits were randomly divided into six groups. The animals were anesthetized by injecting 3% pentobarbital sodium and then fixed on an operating table. The heart was exposed through a thoracotomy, and the pericardium was removed with fine forceps. A 0.9 mm inner diameter needle (XB‐ZT0313, Xiangbo Biotechnology) was used to pierce the ventriculus sinister of the rabbit hearts. To create a hemostatic sealing, the preweighed carbonized mushroom aerogels, Gauze, and gelatin sponges with fixed shapes were applied to the bleeding site. The livers without any treatment were set as the blank control group. The total blood loss and the time to hemostasis were recorded. After confirmation of hemostatic sealing, the chest was closed. The rabbit hearts were collected for histological analysis after implantation for 7 days and 14 days.

### Histological Analysis

After implantation for 7 days and 14 days, the collected tissue samples were fixed in 4% paraformaldehyde, embedded in paraffin, and submitted for histological processing and H&E staining.

### Biosafety Evaluation In Vivo

The subcutaneous implantation test was conducted to assess the biocompatibility of the carbonized mushroom aerogels in vivo. The rats were anesthetized by injection of 3% pentobarbital sodium. A 1‐cm incision was made on the back of the rats. The different carbonized mushroom aerogels were implanted into the incision. After 3, 14, and 70 days, rats were sacrificed, and the injured tissue in contact with the carbonized mushroom aerogels was collected. The collected samples were fixed in 4% paraformaldehyde, embedded in paraffin, and sectioned into slices with 5 µm. The potential detrimental inflammatory response was analyzed by H&E staining.

### Statistical Analysis

Statistical results were expressed as mean ± standard deviation (mean ± SD), and all data were statistically analyzed using SPSS 20.0 or GraphPad Prism8 software, and the results were plotted using GraphPad Prism8. All data were analyzed by one‐way ANOVA. *p* < 0.05 was considered statistically significant. Statistical significance was considered at *p < 0.05, **p < 0.01, ***p < 0.001, and ****p < 0.0001.

## Conflict of Interest

The authors declare no conflict of interest.

## Supporting information

Supporting Information

## Data Availability

The data that support the findings of this study are available from the corresponding author upon reasonable request.
