# Supplementary material for: Super‐Elastic Carbonized Mushroom Aerogel for Management of Uncontrolled Hemorrhage
Source: Adv Sci (Weinh). 2023 Apr 10;10(16):2207347. doi: 10.1002/advs.202207347 (PMC10238221; doi:10.1002/advs.202207347)
Supplement: Supplementary file 1 — Supporting Information [file ADVS-10-2207347-s001.pdf]

Super-elastic Carbonized Mushroom Aerogel for Management of Uncontrolled Hemorrhage

Ganghua Yang[§], Zhenzhen Huang[§], Alec McCarthy, Yueyue Huang,
Jingye Pan[*], Shixuan Chen[*], Wenbin Wan[*]

G. Yang, W. Wan
Department of Orthopaedic Surgery, The Second Affiliated Hospital of Nanchang University, Nanchang, Jiangxi 330006, China
Email: zwwb214@163.com

G. Yang, Z. Huang, S. Chen
Zhejiang Engineering Research Center for Tissue Repair Materials, Wenzhou Institute, University of Chinese Academy of Sciences, Wenzhou, Zhejiang 325000, China
E-mail: chensx@ucas.ac.cn

A. McCarthy
Department of Surgery-Transplant and Mary and Dick Holland Regenerative Medicine Program, University of Nebraska Medical Center, Omaha, NE, 68198 USA

Y. Huang, J. Pan
Key Laboratory of Intelligent Treatment and Life Support for Critical Diseases of Zhejiang Province, Wenzhou, Zhejiang 325000, China.
Zhejiang Engineering Research Center for Hospital Emergency and Process Digitization, Wenzhou, Zhejiang 325000, China
Email: panjingye@wzhospital.cn

Agaricus bisporus

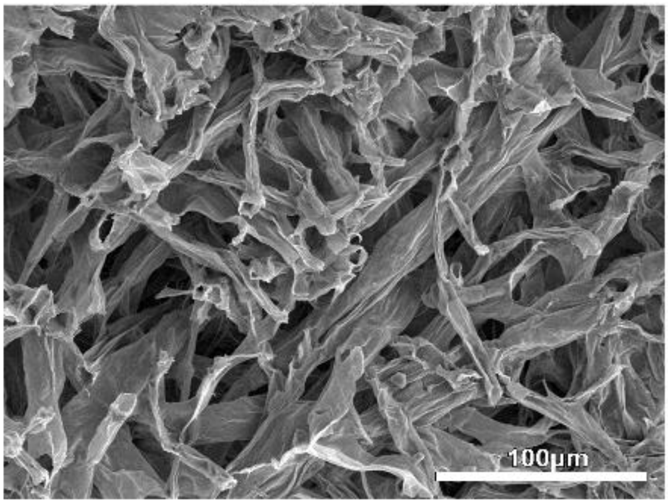

Fig. S1. The natural structure of the *Agaricus bisporus*. SEM images of the internal structure of the *Agaricus bisporus*.

Charcoal

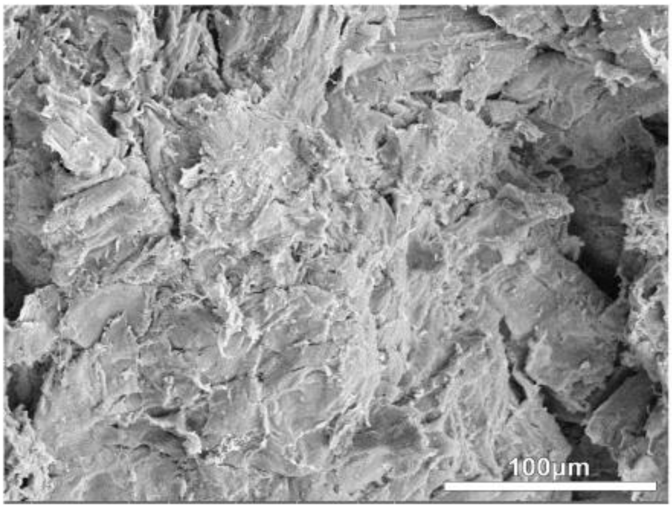

Fig. S2. The structure of the Charcoal. SEM images of the internal structure of the Charcoal.

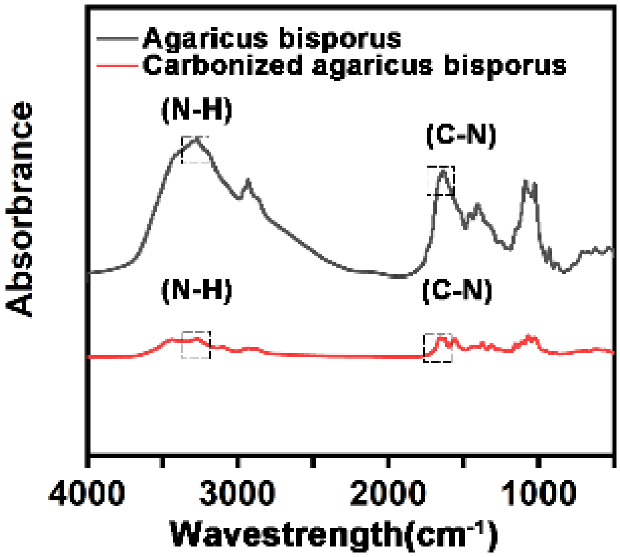

Fig. S3. The chemical functional groups of Agaricus bisporus before and after carbonization. The FTIR spectra, N-H and C-N characterization of Agaricus bisporus before and after carbonization.

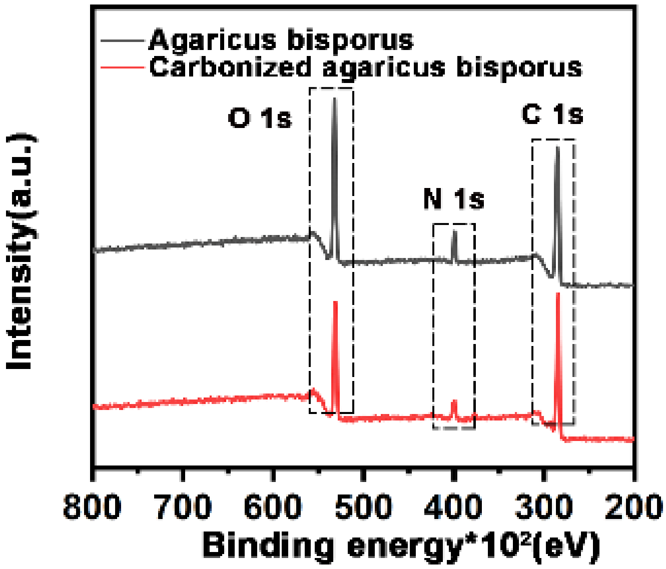

Fig. S4. The chemical functional groups of *Agaricus bisporus* before and after carbonization. The XPS spectra, C, O, and N characterization of *Agaricus bisporus* before and after carbonization.
